# Alert cell strategy in SIRS-induced vasculitis: sepsis and endothelial cells

**DOI:** 10.1186/s40560-016-0147-2

**Published:** 2016-03-23

**Authors:** Naoyuki Matsuda

**Affiliations:** Department of Emergency and Critical Care Medicine, Nagoya University Graduate School of Medicine, Tsurumaicho 65, Showa-ku, Nagoya, 855-4660 Japan

**Keywords:** Sepsis, Endothelial cell, SIVA, Vasculitis

## Abstract

Sepsis refers to systemic inflammatory response syndrome and organ failure resulting from infection. Inflammatory receptors (e.g., Toll-like receptors and nucleotide oligomerization domain) recognize bacterial components as inflammatory ligands. These are expressed not only in leukocytes but also in major organs and vascular endothelial cells. “Alert cell” is defined as the cell that expresses the inflammatory receptor and intracellular signaling system to produce inflammatory mediators such as inflammatory cytokines, chemokines, nitric oxide, and prostanoids in organs and the vasculature. NF-κB and AP-1, which are the transcriptional factors of these inflammatory molecules, are important regulators of multiple organ failure in sepsis and systemic inflammation. The vascular endothelial injury would induce multiple organ failure as tissue ischemia and organ death. Drug discovery targeted at alert cells holds a promise for therapy of inflammation including sepsis.

## Introduction

Sepsis is a systemic inflammatory response syndrome (SIRS) and/or leads to organ dysfunction due to infection. An old definition of sepsis was a SIRS with infection in a joint conference by the American College of Chest Physicians and the Society of Critical Care Medicine in 1991 [1]. The septic clinical outcome has still been bad. In-hospital mortality in septic shock is hardly satisfactory over 20 % with high treatment cost in many countries in 2015. When indirect deaths that complicate chronic conditions (e.g., cardiac failure, cancer) would be included, over 50,000 people a year were estimated to die from sepsis even in Japan. We need to seek the key issue to solve the problem in the clinical management and drug discovery.

In 2001, Angus et al. reported 751,000 deaths across seven states in the USA due to increased severity of sepsis, with a mortality rate of 28.6 % [2]. The “Surviving Sepsis Campaign guidelines” were drafted to improve survival in 2004 [3], with revisions published in 2008 [4] and in 2013 [5]. Hospital mortality rates dropped 0.7 % per site for every 3 months after the participation of the Surviving Sepsis Campaign in 2004 [6]. And in Japan, the Japanese Society of Intensive Care Medicine published the Japanese guidelines [7] for the management of sepsis. However, there was a large difference in the outcome among the facilities in Japan.

There is currently no definitive drug for sepsis and systemic inflammation with multiple organ failure except for antimicrobial agents. This article will provide basic point of view for the clinical management and future drug discovery in sepsis and inflammation.

## Review

### Inflammatory receptors

The role of human Toll-like receptors (TLRs) and the other inflammatory receptors has considerably advanced to know since the several pioneering studies that demonstrated a relationship between Toll receptors and natural immunity [8–11]. TLR, nucleotide oligomerization domain (NOD), and NOD containing leucine-rich repeats (NLRs) are recognized as one of receptors of pathogen-associated molecular patterns (PAMPs) and damage-associated molecular patterns (DAMPs) on microorganisms. The structures of the leucine-rich repeat domain have been determined at NLRC4, NLRP1, and NLRX1 in NLRs and at TLR1-5, TLR6, TLR8, and TLR9 in TLRs. Working out the intracellular signaling pathways downstream of these receptors has provided a clearer picture of how systemic inflammation occurs in sepsis and SIRS conditions [11, 12]. To this end, recent studies have elucidated the signaling pathways downstream of receptors implicated in sepsis, including the TNF receptor (TNF-R) [13, 14] and interleukin receptors (e.g., IL-R1 [15, 16], IL-R6 [17, 18]). These led to a better understanding of mechanisms underlying the propagation and amplification of inflammation between cells after TLR stimulation.

While sepsis therapies have been pursued against many different inflammatory mediators, these mediators can be collectively considered products of transcriptional activation via inflammatory receptor signaling. In particular, the mechanism of amplifying inflammation through the transcription factors such as nuclear factor-κB (NF-κB) and activator protein-1 (AP-1) is particularly important in SIRS-induced vasculitis (SIVA) of vascular endothelium.

From the perspective of gene expression, which adds an additional layer of complexity to sepsis, transcriptional activity of NF-κB and AP-1 was focused in this manuscript. It is considered that the pathophysiology accompanies the increase in sepsis severity at the cellular level and discusses possibilities in new drug discovery.

### The definition of alert cell and SIVA

**Fig. 1:**
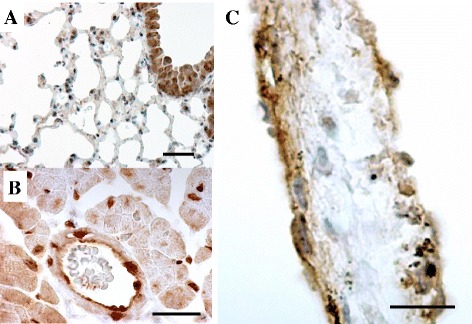
TLR4 expression in the lung, the right atrium, and the atrial septum. The figure is an immunohistochemical staining of Toll-like receptor 4 (TLR4) in the right lower lung (**a**), right atrium (**b**), and atrial septum (**c**) in 12-week-old male BALB-C mice. TLR4 expressed at alveolar type 2 cells, vascular endothelial cells in lung and in cardiomyocytes and vascular endothelial cells in right atrium, and the endocardium of the atrial septum by the stain of 3,3′-diaminobenzidine and tetrahydrochloride with anti-TLR4 antibody. *Bar*: 100 μm

The most important issue to induce multiple organ failure in inflammation is the expression and location of inflammatory receptors (e.g., TLR, TNF-R, and IL-R) and the intracellular signaling molecules on the various epithelial cells and vascular endothelial cells. SIRS pathophysiology was previously considered to result from infiltration of leukocytes, such as neutrophils and dendritic cells, and the overproduction of inflammatory cytokines. However, this theory cannot completely explain why the earliest organ symptoms of SIRS (e.g., acute lung injury and tachycardia) are readily manifest and differ in the extent of inflammation across major organs. Similar to leukocytes, some epithelial cells in major organs express inflammatory receptors on their surface and can produce inflammatory mediators such as inflammatory cytokines, chemokines, nitric oxide (NO), and prostanoids as “inflammatory alert cells” (hereafter, alert cell) shown as Fig. 1 [19] . A 3,3′-diaminobenzidine and tetrahydrochloride staining revealed TLR4 expression in alveolar epithelial type 2 cells, vascular endothelial cells in lung (Fig. 1a) and in cardiomyocytes and vascular endothelial cells in right atrium (Fig. 1b), and the endocardium of the atrial septum (Fig. 1c). The alert cell is defined as the inflammatory-response cell, which can recognize ligands such as DAMPs and PAMPs in tissues and produce chemokine and inflammatory molecules. Vascular endothelial cell is one of the alert cells, which can induce SIVA by recognizing several types of ligands such as DAMPs and PAMPs and producing inflammatory molecules and coagulation factors. The SIVA is defined as an endothelial inflammation in SIRS. Alert cell strategy is a cell-targeting protection therapy for alert cells in systemic inflammation.

To date, many receptors that trigger inflammation have been characterized as described above, including TLR, TNF-R, IL-1R, NOD, NLR, C-type lectin receptor (CLR), receptor for advanced glycation end product (RAGE) [20, 21], and protease-activated receptor (PAR) [22–24]. TLR is recognized as one of the receptors of PAMPs and DAMPs on microorganisms (Table 1). To fully appreciate multiple organ failure associated with SIRS, it is necessary to know which cell types in normal organs express these inflammatory receptor signals, how they are distributed, and how their levels change over the course of SIRS progression. Furthermore, while the early phase inflammatory alarm in response to foreign agents in major organs is carried out by alert cells [12, 25], these cells also increase in number due to SIRS complication or sustained inflammation. Alert cells express inflammatory receptors on their surface and in their cytoplasm and are characterized by the existence of intracellular signaling pathways of these inflammatory receptors. Although vascular endothelial cells may be histologically related, all of them do not have the same biochemical capacity to evoke inflammation under normal conditions as alert cells. While some are sensitive to PAMPs and cytokines, others are not. The histologically related but biochemically distinct cells coexist in the same tissue in major organs including vascular endothelium. When inflammatory stimulation is emerged in response to bacterial infection, the number of alert cells with inflammatory signal increases, thereby accelerating inflammation in major organs.

**Table 1:** Toll-like receptor ligands

TLR subtype	PAMPs	DAMPs
TLR1	(w/TLR2) triacyl lipoprotein	n.d.
TLR2	Lipoproteins (w/TLR1), triacyl lipoprotein (w/TLR6), diacyl lipoprotein, LTA, zymosan	(w/TLR6) HMGB1, HSPs, ECM
TLR3	dsRNA	mRNA
TLR4	LPS, viral envelop proteins	HMGB1, HSPs, ECM, Ox-phospholipids, β-defensin 2 (w/TLR6) Amyloid-β, Ox-LDL
TLR5	Flagellin	n. d.
TLR6	(w/TLR2) diacyl lipoprotein, LTA, zymosan	(w/TLR2) HMGB1, HSPs, ECM
mTLR7/hTLR8	ssRNA	ssRNA (immune complex)
TLR9	DNA, hemozoin	DNA (immune complex)
TLR10	Unknown	n. d.
TLR11	Profilin-like molecule, Uropathogenic bacteria	n. d.

*n. d.* no data

### Transcription factor activation in SIVA

**Fig. 2:**
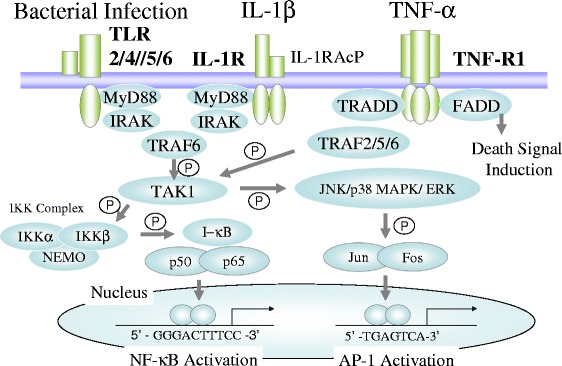
Intracellular signal transduction of inflammatory receptors in alert cells in SIVA. In SIRS-associated vasculitis (SIVA), vascular endothelial cells have an intracellular signal transduction to activate transcriptional factors through inflammatory receptors. Nuclear factor-κB (NF-κB) and activator protein-1 (AP-1) were activated in alert cells of vascular endothelial cells and lead to produce inflammatory mRNA. *TNF-R1 *tumor necrosis factor receptor, *MyD88* myeloid differentiation factor 88, *IRAK* interleukin-1 receptor-associated kinase, *TRAF* tumor necrosis factor (TNF) receptor-associated factor, *TAK1* TGF-β-activated kinase 1, *JNK* c-Jun N-terminal kinase, *ERK* extracellular signal-regulated kinase, *MAPK* mitogen-activated protein kinase, *IKK* inhibitory κB kinase, *NEMO* NF-κB essential modulator, *I-κB* inhibitory-κB, *TRADD* TNF receptor-associated death domain, *FADD* Fas-associated death domain, *p* phosphorylation

Overproduction of inflammatory cytokines and mediators in SIRS and sepsis results from a combination of inflammatory receptor activation by PAMPs and DAMPs and increased messenger RNA (mRNA) production of acute phase response protein [11, 12, 25]. Mechanisms of NF-κB and AP-1 activation have become elucidated in acute phase response and provide a basis for the overproduction of mRNA of acute phase molecules associated with systemic inflammation. The intracellular signaling pathways of the two transcription factors in vascular endothelial cells are depicted in Fig. 2.

In the response, TLR and IL-1R activate TNF-R-associated factor 6 (TRAF6) and tumor growth factor-β-associated kinase 1 (TAK1) via adapter proteins such as myeloid differentiation factor 88 (MyD88) and interleukin-1 receptor-associated kinase (IRAK), especially ILAK1 and ILAK2/4 in vascular endothelial cells. The input signals for this phosphorylation cascade are TLR2 for lipoteichoic acid, TLR4 for LPS, and TLR5 for bacterial flagellin, shown as Table 1. TNF-α and IL-1β are produced during inflammation and hypoxia in the perioperative period and induced similar inflammatory signals as SIRS in alert cells via the respective receptors. As a result, these inflammatory signals induce phosphorylation of the inhibitory-κB (I-κB) kinase (IKK) complex, leading to increased nuclear translocation of NF-κB as the canonical pathway (Fig. 2). NF-κB decoy oligonucleotides reduced inflammation in several tissues in septic mouse study [26–28]. As with NF-κB, AP-1 activity increases in response to mitogen-activated protein kinase (MAPK) activation. Activated via TAK1, MAPK kinase (MAPKK) phosphorylates Jun and Fos family members, which then bind to AP-1 and CRE sites in target promoters.

**Fig. 3:**
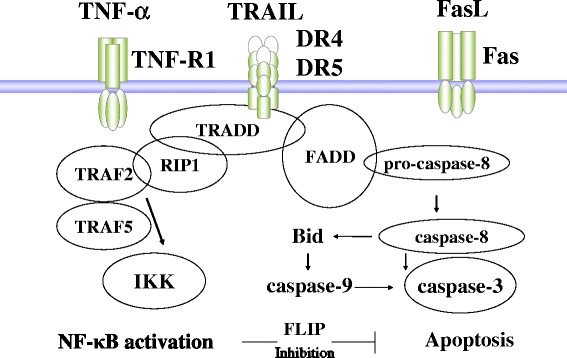
The death receptor signaling in SIVA. Death receptors (*DR*) signal is present also in alert cells of the major organs and vasculature in SIRS-associated vasculitis (SIVA) including sepsis. TNF-R1, Fas (CD95), DR4 (TRAIL receptor 1), and DR5 (TRAIL receptor 2) have a death signal in alert cells via adaptor molecules of TRADD and FADD. On the other side, TNF-R1 produces the anti-apoptotic factors such as FLIP and BclX via activation of NF-κB through RIP1-TRAF pathway. When NF-κB activity decreases and AP-1 activity increases, DR family and FADD tend to proceed apoptosis in alert cells in vascular endothelium. *TNF-R1* tumor necrosis factor receptor 1, *TRADD* TNF receptor-associated death domain protein, *RIP1* receptor-interacting protein 1, *IKK* inhibitory-κB kinase, *TRAF* tumor necrosis factor receptor-associated factor, *FADD* Fas-associated death domain protein, *NF-κB* nuclear factor-κB, *FLIP* FLICE inhibitory protein

Such activation of NF-κB and AP-1 occurs in alert cells of various organs, including vascular endothelial cells, type II alveolar epithelial cells, right atrial cells, renal tubular epithelial cells, and intestinal epithelial cells. In addition to being involved in the NF-κB and AP-1 activation pathway via TNF receptor-associated death domain protein (TRADD), TNF-R can activate death signals via Fas-associated death domain (FADD) in a pathway distinct from TLR and IL-1R in Fig. 3. Alert cells undergo autophagy and apoptosis via increased death receptor (DR) signaling after producing inflammatory mediators to alert surrounding cells in response to inflammatory signals [29–31]. When alert cells increase, and the rate of apoptosis increases relative to that of proliferation, the number of cells that comprise major organs decreases. This subsequently leads to intensification of multiple organ failure. In this context, fibroblast begins to lead to scarring and restriction in organ tissue because of no negative control from alert cells. Fibroblast can be activated into myofibroblasts by adrenergic beta stimulation and thrombin exposure in SIVA.

### NF-κB activation in alert cells

NF-κB is a collective term for the Rel/NF-κB family, which contains a domain homologous to the proto-oncogene c-rel. The Rel/NF-κB family comprises RelA (p65), RelB, c-Rel, NF-κB1 (p105/p50), and NF-κB2 (p100/p52) [32]. These five subunits can homo- or heterodimerize and are prevented in cytoplasm from binding to DNA through interactions with inhibitory κB (I-κB) proteins (i.e., I-κBα, I-κBβ, I-κBε, I-κBγ, I-κBζ, Bcl-3, p105, p100). NF-κB activation occurs through canonical and non-canonical pathways.

TLR signaling in response to infection or inflammation amplifying signals of IL-R and TNF-R ultimately converge on I-κB kinase (IKK) complex phosphorylation and the subsequent decrease in cytoplasmic I-κB levels. The IKK complex exists in the cytoplasm as a large complex of over 700 kDa and is composed of a trimer of IKKα, IKKβ, and IKKγ/NF-κB essential modulator (NEMO). When activated, IKKα phosphorylates serines 32 and 36 of I-κBα and IKKβ phosphorylates serines 19 and 23 of I-κBβ. NEMO can be activated by both phosphorylation and ubiquitination. Upon IKK complex activation and subsequent I-κB phosphorylation, I-κB is poly-ubiquitinated and targeted for proteasome-mediated degradation. The NF-κB dimer is then liberated from the inhibitory complex and translocates into the nucleus, where it binds to its cognate target sequence (GGGACTTTCC) and transcriptionally induces inflammatory cytokines and apoptosis inhibiting factors via the canonical pathway. Signaling through TLR and IL-R also uses the canonical pathway and results in nuclear translocation of the RelA and p50 dimer.

On the other side, a non-canonical NF-κB pathway exists in leukocytes and epithelial cells [33]. In this pathway, IKKα free of NEMO or IKKβ requires phosphorylation by NF-κB inducing kinase (NIK) to become an active form. When the RelB-p100 dimer gets to be phosphorylated by IKKα, p100 undergoes limited processing and generates a RelB-p52 dimer. This dimer then undergoes nuclear translocation and activates transcription of NF-κB target genes. Some studies have shown that the NF-κB dimer is trapped in the nucleus by I-κBζ [34] and that IKKα can localize to both the cytoplasm and nucleus [35]. Nuclear I-κBζ gets phosphorylated by nuclear IKKα, which likely leads to I-κBζ degradation and NF-κB activation. Some studies suggest the possibility that free cytoplasmic RelA can transport IKK into the nucleus by forming a complex. Nuclear IKK can then phosphorylate histone H3 associated with chromatin and increase the transcription of small G proteins and immediate early genes such as Fos and Jun [36]. Given the lack of a full understanding of the non-canonical pathway, various aspects will need to be elucidated in vascular endothelium in future research.

Table 2 lists representative proteins that are overproduced as a result of NF-κB activation in SIRS, shock, and disseminated intravascular coagulation: inflammatory cytokines such as TNF-α, IL-1, and IL-6; inducible nitrogen oxide synthase (iNOS) that produces high levels of nitric oxide (NO); inducible cyclooxygenase-2 (COX2) that leads to production of prostanoids such as prostacyclin; granulocyte-macrophage colony-stimulating factor (GM-CSF), granulocyte colony-stimulating factor (G-CSF), and macrophage colony-stimulating factor (M-CSF), which accelerate the move of immature granulocytes and macrophages to peripheral and have a protective effect in vascular endothelial cells; chemokines and adhesion molecules that function in leukocyte migration and infiltration in vascular endothelium; coagulation molecules von Willebrand factor and tissue factor; and plasminogen activator inhibitor-1 as fibrinolysis inhibitor. These productions depend partly on NF-κB activation in SIRS and sepsis. In order to understand systemic inflammation, it is key to note that NF-κB activation underlies the generation of inflammatory cytokines and inflammatory molecules.

**Table 2:** Inflammatory gene expression after nuclear factor-κB activation

Proinflammatory cytokines	Inflammatory enzymes
Tumor necrosis factor α	Inducible nitric oxide synthase
Interleukin-1β	Inducible cyclooxygenase-2
Interleukin-2	5-lipoxygenase
Interleukin-6	Adhesion molecules
Chemokines	Intracellular adhesion molecule-1
Interleukin-8	Vascular cell adhesion molecule-1
Macrophage inflammatory protein 1α	E-selectin
Macrophage chemotactic protein 1	Coagulation
Eotaxin	von Willebrand factor
Gro-α, Gro-β, Gro-γ	Tissue factor
ΕΝΑ–78	Plasminogen activator inhibitor-1

**Fig. 4:**
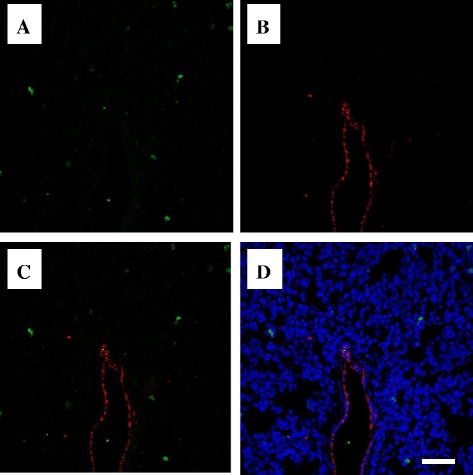
Expression of the glucocorticoid receptor in the guinea pig lung. **a** Glucocorticoid receptor α (FITC-chromogenic). **b** Vascular endothelium (anti-von Willebrand factor antibody and Cy3-chromogenic). **c** The superposition image of **a** and **b**. **d** The superposition image of **c** and nuclear staining by Hoechst. Glucocorticoid receptor α was not abundantly expressed in lung vasculature. *Bar*: 100 μm

Glucocorticoids (e.g., prednisolone, methylprednisolone), which were conventionally used to reduce inflammation in rheumatoid arthritis, exert a NF-κB inhibiting effect via glucocorticoid receptor α (GRα). While inflammatory infiltration cells (e.g., monocytes, neutrophils, and lymphocytes) express GRα, few expression has been observed on alert cells of major organs and vascular endothelium [36]. Because alert cells are known to express scavenger receptors such as CLR and LOX-1 on their surface in response to NF-κB activation, the success rate of gene therapy by liposome encapsulation markedly increases [25–30]. While glucocorticoids mainly target cells involved in fresh cell-mediated immunity, such as immature cells, lymphocytes, and dendritic cells that express GRα, several types of decoy oligonucleotide aim to reduce the induction of inflammation by alert cells (Fig. 4). From this perspective, studies have shown that while NF-κB decoys effectively reduce inflammation in major organs in sepsis, they also induce apoptosis [25–30]. This cell targeting therapeutic concept was defined as “alert cell strategy” for vascular endothelial cells in this article.

### NF-κB activation in SIVA

Apoptosis during the early stages of inflammation is prevented by NF-κB-mediated production of anti-apoptotic molecules such as FLICE-inhibitory protein (FLIP), inhibitors of apoptosis (IAPs), and BclX in alert cells [37, 38]. In SIRS and sepsis, the main factor leading to apoptosis in vascular endothelial cells is the enhanced expression of death receptor (DR) family and the downstream death adapter proteins [29–31] . As established inducers of apoptosis, TNF-R1, CD95 (Fas), DR4 (TRAIL receptor 1), and DR5 (TRAIL receptor 2) are expressed not only in immunocompetent cells but also in major organs and vascular endothelial cells. These DR family members form a complex with Fas-associated death domain (FADD) protein, procaspase-8, procaspase-10, and c-FLIP to generate the death-inducing signal complex (DISC) (Fig. 3). Although c-FLIP, which inhibits procaspase-8 processing, increases in response to NF-κB activation, it decreases as sepsis progresses due to the associated reduction in NF-κB activity [37]. The transcriptional induction of DR family members and FADD in the lungs and vascular endothelial cells in sepsis has been confirmed by RT-PCR and Western blot analysis. The activation of caspase 3 following DISC activation leads to apoptosis [28–30].

**Fig. 5:**
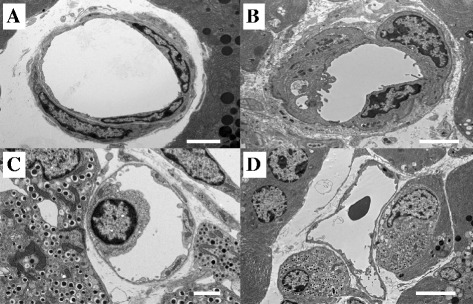
Transmission electron microscope image of a mouse pancreas capillary vessel after cecum ligation and puncture. The figure was a transmission electron microscope image in septic mouse pancreatic capillaries. A male BALB-C mouse was ligated approximately 5 mm of the cecal tip and penetrated through by 23G needle (*CLP*cecum ligation and puncture). **a** Normal. **b** Septic 12 h. **c**, **d** Septic 24 h. In the time course after CLP, vascular endothelial cells were bulging and swelling and dropped off the basement membrane of vascular endothelium. *Bar*: 20 μm

Sepsis can be induced in male BALB-C mice by 6-mm skin incision and ligating the cecum at 5 or 10 mm from the distal end, followed by cecal puncture with a 21G or 23G needle in the original method called a keyhole cecal ligation and puncture (keyhole-CLP) [27, 29, 31]. These CLP mice die within 2 days of the procedure and show two peaks of NF-κB activation at around 10 and 15 h, which is followed by a progressive decrease in activity. A layer of vascular endothelial cells is found on the surface of an approximately five-layered smooth muscle layer of the mouse aorta, which does not exhibit swelling or shedding under normal conditions. Yet, as sepsis progresses, vascular endothelial cells tend to swell and induce cell death (Fig. 5). In addition to immunocompetent cells, alert cells in vascular endothelium are also affected by autophagy and apoptosis. Caspase inhibitors, FADD inhibitors, and gene therapy using siRNAs targeting these factors that aim to reduce apoptosis following inflammation hold promise as potential therapies [29–31].

### AP-1 activation in SIVA

AP-1 is activated in alert cells under severe conditions associated with SIRS and sepsis. While AP-1 functions in cell proliferation and differentiation, it also induces excessive synthesis of catecholamines in the central nervous system and apoptosis of alert cells in the periphery [39–41].

AP-1 genes consist of four families: Jun, Fos, activating transcription factor (ATF), and the musculoaponeurotic fibrosarcoma (MAF) oncogene. Jun homodimers or Jun/Fos heterodimers recognize and bind to the phorbol 12-*O*-tetradecanoylphorbol-13-acetate-responsive element (TPA-responsive element; TRE), and Jun/ATF complexes bind to the cAMP-responsive element (CRE), leading to the transcriptional induction of inflammatory markers (COX-2, ICAM-1), matrix metalloproteinases (MMP-1, MMP-3, MMP-9), and proteins involved in actin polymerization (CapG, Ezrin, Krp-1, Mts-1). These signal transduction are observed not only in vascular endothelial cells but also in fibroblasts in vascular formation.

MAPK is a serine/threonine kinase comprised of four subfamilies: extracellular signal-regulated kinase (ERK), Jun-N-terminal protein kinase (JNK), p38 MAPK, and ERK5/big MAPK1 (BMK1). In SIVA, MAPK is activated in response to TAK-1 activation in alert cells. This leads to the subsequent transcriptional activation of AP-1 targets. JNK is activated during sepsis and phosphorylates the N-terminal transcriptional activation domains (TADs) of Jun family members, such as c-Jun, JunB, and JunD. ERK phosphorylates the TADs of Fos family members, such as c-Fos, FosB, Fra-1, and Fra-2. These phosphorylation events induce the heterodimerization of Jun and Fos family members or stabilize Jun homodimers, resulting in increased binding to the AP-1 binding site TRE (TGACTCA). While c-Jun, c-Fos, and FosB have N-terminal TADs, the TADs of JunB, JunD, Fra-1, Fra-2, and FosB2 are weak. As such, they are weak AP-1 transcriptional activators. With respect to ATF family members (e.g., ATF-2, ATF-3, B-ATF), phosphorylation by JNK or p38MAPK leads to their homodimerization or heterodimerization with Jun family members and increased binding to CRE sites (TGACGTCA). AP-1 activation tends to be delayed compared to NF-κB activation. One likely reason for this is that the temporary activation of phosphatases that inhibit MAPK (e.g., protein phosphatase 2A, protein phosphatase 2C, and MAPK phosphatase) is carried out by MAPK itself. AP-1 can be considered an exacerbating factor in SIVA that contributes to increased death signaling in alert cells and to decrease fibroblast formation.

### KLF

Kruppel-like transcriptional factor (KLF) is a transcription factor of the zinc finger family that has recently gained attention for its role in reducing inflammation in vascular endothelial cells and inducing endothelial NO synthetase (eNOS) and thrombomodulin expression in these cells [42, 43]. KLF2 exhibits anti-inflammatory effects by inhibition of thrombin receptor PAR1 in vascular endothelial cells [43]. Its role in inhibiting AP-1 and NF-κB activity in vascular endothelial cells has also been reported [44–48].

In early stages of SIVA, iNOS expression increases in blood vessels, while eNOS expression decreases [49]. Distributive warm shock results when blood vessels subsequently become dilated and exhibit impaired blood flow regulation due to shear stress. A potential mechanism that underlies the simultaneous increase in iNOS and decrease in eNOS involves the nuclear translocation of NF-κB [25, 49] and the methylation of histone in the gene promoter region of eNOS [50]. In this mechanism, nuclear transferred NF-κB mediates the overproduction of iNOS mRNA, as well as histone acetylation, which consequently leads to a decrease in KLF2 and KLF4 transcripts and eNOS expression. Statins can recover decreased eNOS levels in SIVA, and this may be related to the ability of statins to increase KLF expression in vascular endothelial cells [43–48]. Statins may have potential applications in the clinical setting for SIVA management. And more, mRNA induction of KLF2 and KLF4 as alert cell strategy may have a protection against SIVA and bring long term surviving in systemic inflammatory patients.

### Molecular markers in SIVA

In cell culture evaluation of vascular endothelial cells using the human umbilical vein endothelial cell (HUVEC) and the human pulmonary arterial endothelial cell (HPAEC), the transcriptional activity was valuable in the main intracellular signaling molecules after an exposure of TNFα 100 ng/mL by an inner control of actin mRNA (Tables 3 and 4). There was a significant deference in mRNA expression between in HUVEC and in HPAEC in the time course after TNFα exposure. Enzyme-linked immunosorbent assay detects the proteins, which mRNA increased at even 4 h after exposure of TNFα, in the culture medium. ILAK-1, TNFRSF1A, c-FOS, c-Jun, and ATG12 were abundantly increased and could be detected in the culture medium of HUVEC and HPAEC. This observation reveals that there could be a possibility to detect them as a cytotoxic molecular marker in SIVA. It seems to be worth to evaluate them in clinical specimens.

**Table 3:** mRNA expression after TNF stimulation in human pulmonary artery endothelial cells

	0 min	15 min	30 min	1 h	4 h	8 h
ATG5	1	0.3	0.3	1.2	0.1	0.2
ATG8	1	4.1	4.5	4.0	5.5	0.6
ATG12	1	6.5	4.9	4.3	6.8	2.2
c-FOS	1	10.3	18.6	6.2	3.1	2.8
c-Jun	1	3.2	8.7	4.4	2.1	0.8
ERK1	1	1.6	1.1	0.6	0.5	0.6
FADD	1	1.8	3.2	3.6	2.8	1.2
ILAK1	1	7.3	12.6	10.3	10.1	2.9
ILAK2	1	2.1	1.8	1.6	1.2	0.5
ILAK4	1	2.2	1.8	1.6	1.4	0.6
MAPK14	1	3.8	3.6	3.1	3.5	1.4
MyD88	1	2.1	1.5	1.3	1.1	0.2
p62	1	5.8	6.8	7.2	6.8	2.1
p65	1	1.1	0.8	0.6	0.4	0.2
RIPK	1	3.3	2.2	1.8	2.4	0.8
STAT3	1	6.3	2.4	2.8	3.3	0.5
TLR2	1	5.8	3.6	5.3	3.6	1.2
TLR4	1	0.7	0.8	0.5	0.6	0.2
TAK1	1	3.8	4.3	3.7	3.6	1.2
TNFRSF1A	1	1.9	2.2	1.9	2.1	1.4
ULK1	1	1.5	1.2	1.3	1.8	0.9

Human pulmonary artery endothelial cells (HPAEC) were observed in the time course in a separate medium after TNFα 100 ng/mL administration without steroid and the other anti-inflammatory molecules. At 8 h after TNFα stimulation, the endothelial cells were observed with shape change and cell death in approximately 35 %. High expression of mRNA of ATG8, ATG12, ILAK1, MAPK14, p62, TLR2, and TAK1 were observed in HPAEC through the time course after TNFα by reverse transcription polymerase chain reaction of the inner control of actin mRNA

**Table 4:** mRNA expression after TNF stimulation in human umbilical vein endothelial cell

	0 min	15 min	30 min	1 h	4 h	8 h
ATG5	1	0.5	0.5	0.7	0.3	0.1
ATG8	1	2.3	1.8	2.6	2.1	0.3
ATG12	1	2.2	3.9	3.1	2.2	0.8
c-FOS	1	13.6	22.9	5.3	2.8	1.3
c-Jun	1	4.6	12.5	4.9	1.1	0.8
ERK1	1	1.2	0.8	0.7	1.0	0.5
FADD	1	2.2	3.1	3.8	1.8	1.1
ILAK1	1	8.8	14.2	12.4	11.0	3.9
ILAK2	1	2.1	1.8	1.6	1.2	0.7
ILAK4	1	2.2	1.8	1.6	1.4	0.5
MAPK14	1	4.2	3.4	3.4	3.9	1.5
MyD88	1	2.2	2.1	1.7	2.1	0.3
p62	1	1.3	3.5	9.4	7.7	1.6
p65	1	0.8	1.4	1.4	1.1	0.5
RIPK	1	3.2	2.7	2.2	3.4	0.8
STAT3	1	6.3	2.4	2.8	3.0	0.2
TLR2	1	6.1	2.8	5.3	2.6	0.4
TLR4	1	1.6	1.3	1.7	1.5	0.2
TAK1	1	2.8	3.9	3.1	3.6	0.8
TNFRSF1A	1	12.4	12.1	12.2	12.3	3.1
ULK1	1	3.7	4.7	2.9	5.1	1.8

Human umbilical vein endothelial cells (HUVEC) were observed in the time course in a separate medium after TNFα 100 ng/mL administration without steroid and the other anti-inflammatory molecules. At 8 h after TNFα stimulation, the endothelial cells were observed with shape change and cell death in approximately 32 %. High expression of mRNA of ILAL1, p62, TNFRF1A, and ULK were observed in HUVEC through the time course after TNFα by reverse transcription polymerase chain reaction of the inner control of actin mRNA

On the other hand, a control HUVEC stimulated with TNFα could be useful to detect circulating endothelial cells in disseminated intravascular coagulation (DIC) and SIVA [51]. Normal vascular endothelial cells are different from inflammatory circulating endothelial cells in the expression of surface membrane molecules. The criteria for a detection of circulating vascular endothelial cells in SIVA need the usage of the vascular endothelial cells subjected to inflammatory stimuli, such as TNFα. This element could be one of the detecting systems in a progression of SIVA. Endothelial cell injury and the detachment from basement membrane would induce the two aspects of “warm shock” and “cold shock” in the microcirculation [52]. Clinical evaluation of long-term prognosis for the complication of SIVA seems to be one of the topics for future research.

## Conclusions

This article approached the topic of multiple organ dysfunction and DIC in SIRS and sepsis from the viewpoint of transcription activity of mRNA in vasculature as SIVA. Inflammation is exacerbated by the increased response of alert cells in vascular endothelium with sepsis progression. Major organs become impaired in function of alert cells as increasing cell death. In vasculature, endothelial cell death would change “warm shock” to “cold shock” in septic shock. Enough blood flow would be impaired in organs. A theory of rescuing alert cells from inflammation and cell death during and following inflammation is called the alert cell strategy. DR signaling and the transcriptional regulation are likely to become a crucial target of vascular resuscitation in prolonged sepsis management.
